# Low serum level of 25‐OH vitamin D relates to Th17 and treg changes in colorectal cancer patients

**DOI:** 10.1002/iid3.723

**Published:** 2022-10-26

**Authors:** Bai Chen, Liugen Jin

**Affiliations:** ^1^ Department of Gastroenterology the Affiliated Hospital of Jiangnan University Jiangsu Wuxi China

**Keywords:** 25(OH)D, colorectal cancer, Th17, Treg

## Abstract

**Background:**

Serum 25‐hydroxyvitamin D [25(OH)D] level alters in colorectal cancer (CRC) development. Regulatory T (Treg) cells and T‐ helper type 17 (Th17) cells are involved in immune response. Th17‐mediated proinflammatory responses contribute to tumorigenesis, and Treg plays different roles in different periods of CRC. Vitamin D deficiency is associated with significant variations in peripheral immune cells. This study investigated the relationship between Th17 and Treg cells and 25(OH)D level in CRC.

**Methods:**

Ninety‐five CRC patients were included, as well as 80 healthy controls during the same period at the Affiliated Hospital of Jiangnan University. 25(OH)D level was analyzed through electrochemiluminescence (ECLIA). Th17 and Treg levels were evaluated through flow cytometry. Serum levels of interleukin (IL)‐10, IL‐17, IL‐23, and transforming growth factor‐β (TGF‐β), were analyzed through commercial enzyme‐linked immunoassay (ELISA) kits.

**Results:**

25(OH)D levels were downregulated in the serum of CRC patients. Decreased 25(OH)D level contributed to CRC pathogenesis. Decreased 25(OH)D level in CRC correlated with increased Treg and Th17 cell ratios and TGF‐β1, IL‐10, IL‐17, and IL‐23 levels in peripheral blood.

**Conclusion:**

Decreased 25(OH)D level in the serum of CRC patients had negative correlation with Treg and Th17 ratios and relative cytokines levels.

## Graphical abstract

1. 25‐hydroxyvitamin D (25(OH)D) levels were downregulated in the serum of colorectal cancer (CRC) patients. 2. Decreased 25(OH)D level contributed to CRC pathogenesis.

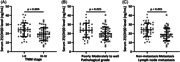

## 1 INTRODUCTION

In China, estimated colorectal cancer (CRC) deaths increased from 111.41 thousand in 2005 to 178.02 thousand in 2020; age‐standardized mortality rate decreased from 10.01 per 100,000 in 2005 to 9.68 per 100,000 in 2020.^1^ Vitamin D level is inversely associated with CRC development, influences prognosis, and survival rate.^2^ In CRC patients undergoing surgery, vitamin D influences the risk of infectious complications.^3^ Higher vitamin D level also contributes to the effect of neoadjuvant treatment.^4^ 25‐hydroxyvitamin D [25(OH)D] level is significantly decreased in CRC patients, and the deficiency and insufficiency of vitamin D are common.^5^ High‐level plasma 25(OH)D has been associated with lower CRC incidence and mortality.^6^

Patients with CRC often have enhanced immune cell infiltrating and immune response contributes to the development of CRC.^7^ Immunotherapy is considered to be effective in the improvement of the prognosis of CRC patients. The association between the 25(OH)D level and CRC patient survival is stronger for carcinomas with lower peritumoural lymphocytic reaction.^6^ Vitamin D deficiency is associated with significant variations in peripheral immune cells.^8^ Regulatory T (Treg) cells and T‐ helper type 17 (Th17) cells are involved in immune response.^9^

Currently, there is still controversy about the role of Th17 in CRC. The mainstream view is that Th17 cells contribute to CRC development.^10^ Th17‐mediated proinflammatory responses contribute to tumorigenesis, and Treg plays different roles in different periods of CRC. Miteva et al. found that Th17 and Treg‐related genes were highly expressed in CRC tumors and local lymph nodes, and related molecules interleukin (IL)‐10, IL‐17, and transforming growth factor‐β (TGF‐β) were all highly expressed.^11^ Another study also found the increase of Treg and Th17 cells during CRC, and the increase of Thl7 occurred in the early stage, while the increase of Treg occurred in the late stage.^12^ This study investigated the relationship between Treg and Th17 cells and 25(OH)D in CRC patients.

## 2 METHODS

### 2.1 Patients

In this study, CRC patients were newly diagnosed with adenocarcinoma of the colon and/or rectum and histopathologically confirmed at the Affiliated Hospital of Jiangnan University. A total of 95 CRC patients were included, as well as 80 healthy controls (HCs) during the same period. This study was approved by the Ethics Committee of the Affiliated Hospital of Jiangnan University (#CI‐hj687). All written informed consents were obtained.

Patients with colorectal cancer are diagnosed by pathological tissue examination through colonoscopy biopsy or surgery and are excluded from anal canal cancer, colorectal polyposis, familial adenomatous polyposis, and other diseases. Clinical staging of colorectal cancer is performed with reference to the International Union Against Cancer (UICC) staging system (2009) and the 7th edition of the American Joint Committee on Cancer.

Exclusion criteria: 1. Patients who are administrated with drugs affecting calcium, phosphorus, and vitamin D levels within 1 month before enrollment, such as vitamin D, estrogen or its analogues, and calcitonin; 2. Patients have hyperparathyroidism or receive parathyroidectomy; 3. Patients have diseases affecting vitamin D absorption, such as diarrhea, chronic pancreatitis, biliary obstruction, colitis, and partial resection of the small intestine; 4. Patients have severe liver and kidney disease, severe peripheral or central nervous lesions, or serious comorbidities (such as heart failure and pneumonia); 5. Patients have fasting or parenteral nutrition.

Healthy controls were tested by chest fluoroscopy, color Doppler ultrasound diagnostic system, electrocardiogram, and several clinical tests (including routine blood, urine, stool + occult blood, blood glucose, blood lipids, liver and kidney function, and tumor markers) and were free of anal diseases and other diseases as confirmed by doctors.

### 2.2 Flow cytometry

A total of 12 ml of whole blood was collected and anticoagulated with ethylenediaminetetraacetic acid dipotassium (EDTA‐K2), of which 6 ml was used for the isolation and identification of Th17 and Treg cells.

Peripheral blood mononuclear cells (PBMCs) were isolated by Ficoll‐Paque (General Electric) and cultured in RPMI 1640 (Invitrogen) with 10% fetal bovine serum (Gibco) and 100 U/ml penicillin/streptomycin (Sigma‐Aldrich). PBMCs were harvested and incubated with anti‐CD4 APC‐Cy7 (4 µl for 10^6^ cells, ab233298), anti‐CD127 FITC (1/700, ab255816), anti‐CCR6 APC (1/3000, ab288297), anti‐CD25 PE (1/1000, ab283576), and anti‐CXCR3 PE‐Cy7 (1/500, ab259865) (Abcam) and sorted by FACSAria cytometer.

### 2.3 Evaluation of 25(OH)D level

Whole blood (12 ml) was collected and anticoagulated with ethylenediaminetetraacetic acid dipotassium (EDTA‐K2), of which 6 ml was used for this assay. 25(OH)D level was analyzed by electrochemiluminescence (ECLIA) using the Elecsys system (Roche). Normal level: ≥30 ng/ml. Insufficiency: ≤20 ng/ml and <30 ng/ml. Deficiency: <20 ng/ml.

### 2.4 ELISA

A total of 12 ml of whole blood was collected and anticoagulated with ethylenediaminetetraacetic acid dipotassium (EDTA‐K2), of which 6 ml was used for the evaluation of cytokine levels. TGF‐β1, IL‐10, IL‐17, IL‐6, and IL‐23 levels were analyzed through commercial ELISA kits (Fine Test).

### 2.5 Statistical analysis

Data were shown as mean ± standard deviation (SD) and analyzed by Fisher's exact test or Mann–Whitney test. Linear correlations were verified by Spearman's correlation analysis. SPSS Statistics 24.0 was employed for statistical analysis. *p* < .05 was thought to be statistically significant.

## 3 RESULTS

### 3.1 25(OH)D levels were downregulated in CRC patients

**Figure 1:**
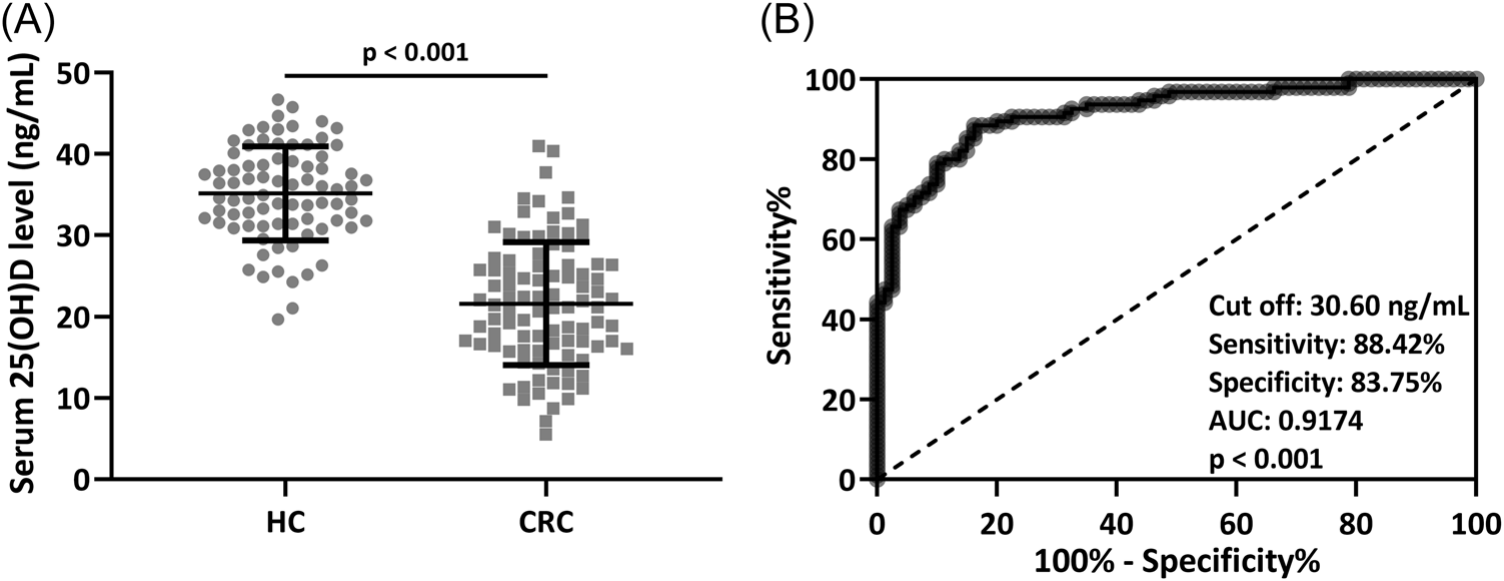
Serum 25‐hydroxyvitamin D [25(OH)D] levels were downregulated in colorectal cancer patients (A, *n* = 80 for HC and *n* = 95 for colorectal cancer [CRC]) and receiver operating characteristics (ROC) analysis of serum 25(OH)D levels (B). Data were shown as mean ± SD. Mann–Whitney test.

As shown in Figure 1A, CRC patients had dramatically lower 25(OH)D levels (35.15 ± 5.79 ng/ml for HC and 21.59 ± 7.54 ng/ml for CRC). Based on the receiver operating characteristics (ROC) curve analysis, the area under curve (AUC) was significant for 25(OH)D (*p* < .001, AUC: 0.92, sensitivity: 88.42%, specificity: 83.75%) and the cut off value was 30.60 ng/ml (Figure 1B).

### 3.2 Decreased 25(OH)D level had correlation with enhanced CRC pathogenesis

Table 1 showed CRC patients' characteristics. Patients were divided into high level group (>20 ng/ml; *n* = 52) and low‐level group (<20 ng/ml; *n* = 43). No significant difference was found in age, gender, and tumor location (all *p* > .05). Significant differences were found in pathological grade (*p* = .014), TNM stage (*p* = .023), and lymph node metastasis (*p* = .013).

**Table 1:** Serum 25(OH)D level and clinicopathological factors in colorectal cancer patients (*n* = 95)

Variables	Serum 25(OH)D level		*p* value
	<20 ng/ml (*n* = 43)	>20 ng/ml (*n* = 52)	
Age (years)			
<60	18	28	.304
≥60	25	24	
Gender			
Male	24	35	.292
Female	19	17	
Tumor location			
Colon	27	37	.511
Rectum	16	15	
TNM stage			
I–II	14	30	.023
III–IV	29	22	
Pathological grade			
Poorly differentiated	13	29	.014
Moderately to well differentiated	30	23	
Lymph node metastasis			
Nonmetastasis	15	32	.013
Metastasis	28	20	

*Note*: Fisher's exact test.

Abbreviation: 25(OH)D, 25‐hydroxyvitamin D.

**Figure 2:**
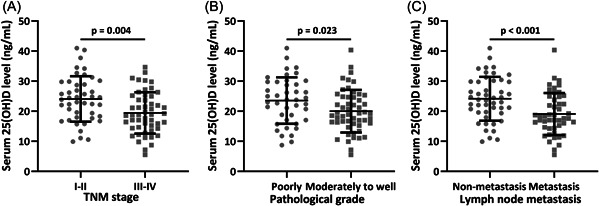
Comparisons of serum 25‐hydroxyvitamin D [25(OH)D] levels between TNM stages of I–II and III–IV (A, *n* = 44 for I–II and *n* = 51 for III–IV), pathological grades of poorly differentiated and moderately to well differentiated (B, *n* = 42 for poorly and *n* = 53 for moderately to well), lymph node nonmetastasis and metastasis (C, *n* = 47 for nonmetastasis and *n* = 48 for metastasis). Data were shown as mean ± SD. Mann–Whitney test.

CRC patients in TNM stages of I–II had significantly higher 25(OH)D levels than those in TNM stages of III–IV (Figure 2A, 24.08 ± 7.56 ng/ml for I–II and 19.44 ± 6.89 ng/ml for III–IV, *p* = .004). CRC patients on pathological grades of poorly differentiated had significantly higher 25(OH)D levels than those on pathological grades of moderately to well differentiated (Figure 2B, 23.57 ± 7.72 ng/ml for poorly and 20.02 ± 7.07 ng/ml for moderately to well, *p* = .023). CRC patients with lymph node metastasis had significantly lower 25(OH)D levels than those with nonmetastasis (Figure 2C, 24.14 ± 7.31 ng/ml for nonmetastasis and 19.09 ± 6.97 ng/ml for metastasis, *p* < .001).

### 3.3 25(OH)D level inversely associated with the ratio of treg and Th17 cells

**Figure 3:**
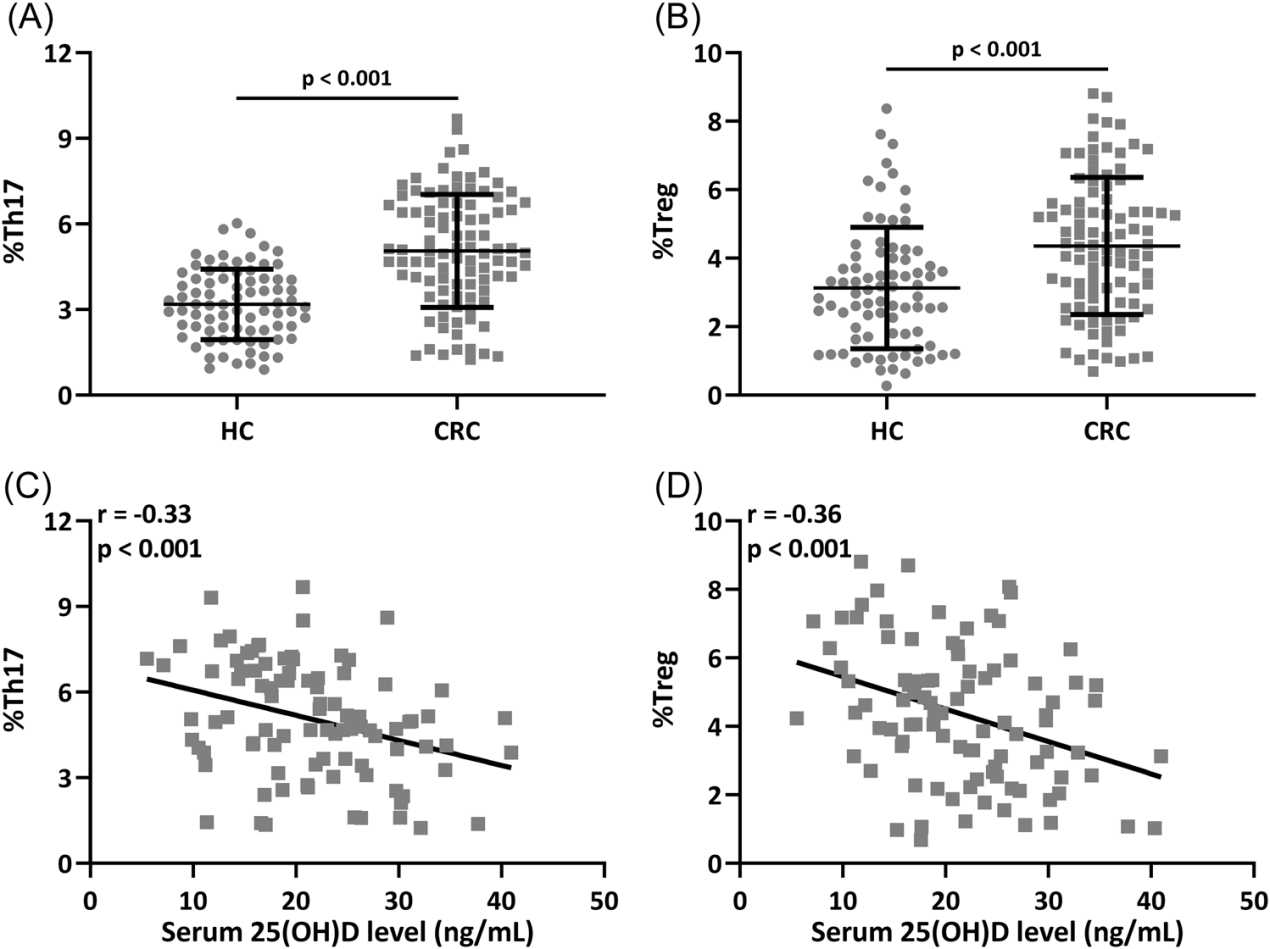
Flow cytometry analysis of Th17 (A) and Treg (B) lymphocyte subpopulations in peripheral blood from healthy controls and colorectal cancer (CRC) patients. *n* = 80 for HC and *n* = 95 for CRC. Data were shown as mean ± SD. Mann–Whitney test. The correlation analysis of serum 25‐hydroxyvitamin D [25(OH)D] level and Th17 (C) and Treg (D) lymphocyte subpopulations in peripheral blood from CRC patients, *n* = 95.

Th17 and Treg cell ratios were significantly upregulated in CRC patients (Figure 3A 3.18 ± 1.24 for HC and 5.06 ± 1.98 for CRC, *p* < .001; and 3B 3.13 ± 1.77 for HC and 4.36 ± 2.01 for CRC, *p* < .001). Representative flow cytometry images of Th17 and Treg lymphocyte subpopulations in peripheral blood from HCs and CRC patients were shown in Supporting Information: Figure S1. 25(OH)D level had negative correlation with Th17 cell ratio (*r* = −.33, *p* < .001) and Treg cell ratio (*r* = −.36, *p* < .001) (Figure 3C,D). IL‐6 is a key player in Th17/Treg balance.

### 3.4 25(OH)D level inversely associated with cytokine levels

**Figure 4:**
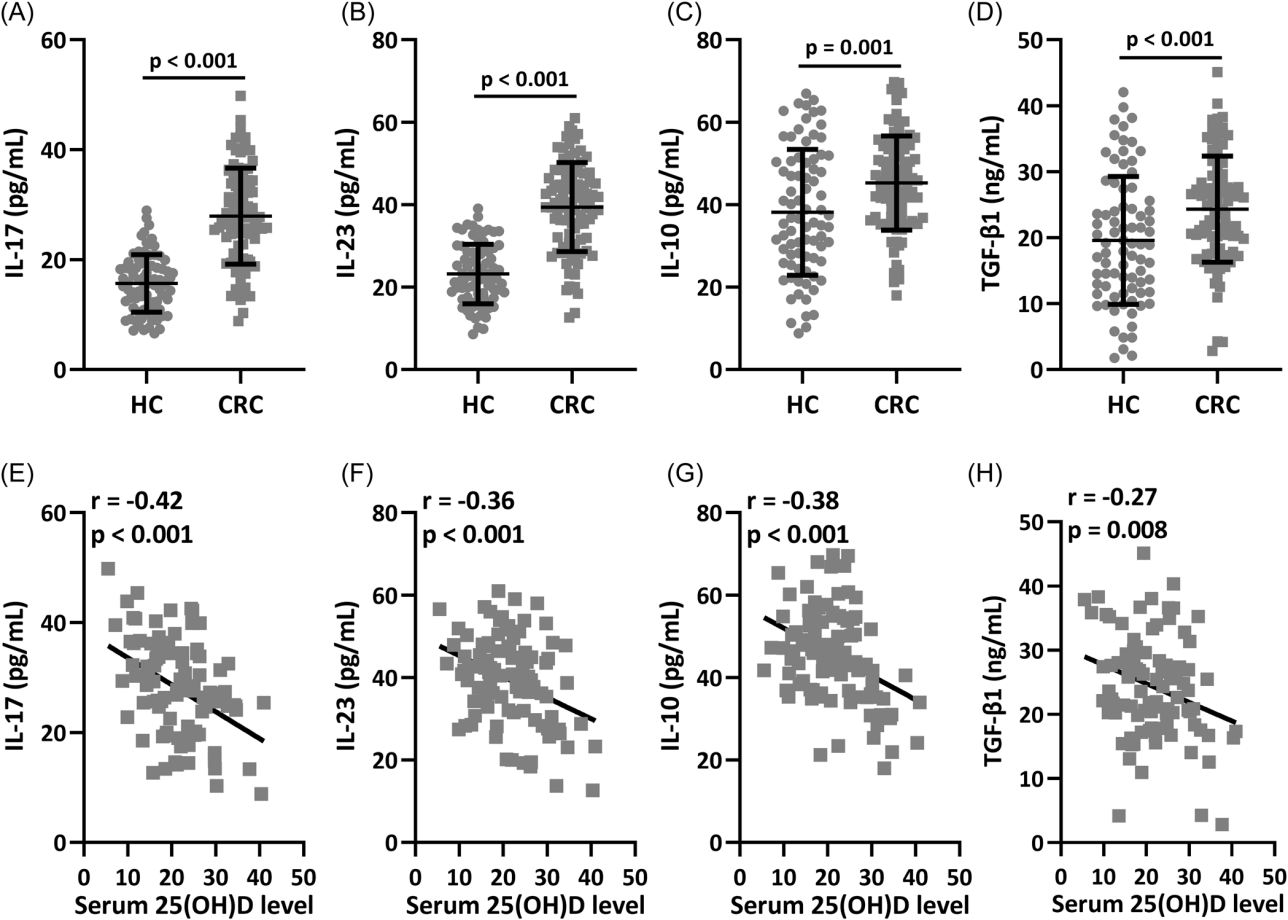
Enzyme‐linked immunoassay (ELISA) analysis of interleukin (IL)‐17 (A), IL‐23 (B), IL‐10 (C), and transforming growth factor‐β (TGF‐β1) (D) in serum from healthy controls and CRC patients. *n* = 80 for HC and *n* = 95 for colorectal cancer (CRC). Data were shown as mean ± SD. Mann–Whitney test. The correlation analysis of serum 25(OH)D level and IL‐17 (E), IL‐23 (F), IL‐10 (G), and TGF‐β1 (H) in serum from CRC patients, *n* = 95.

ELISA results demonstrated that the levels of TGF‐β1, IL‐10, IL‐23, and IL‐17 were significantly higher in CRC patients (Figure 4A, 15.67 ± 5.22 for HC and 27.91 ± 8.75 for CRC, *p* < .001; 4B 23.18 ± 7.19 for HC and 39.42 ± 10.79 for CRC, *p* < .001; 4 C 38.15 ± 15.27 for HC and 45.27 ± 11.41 for CRC, *p* = .001; 4D 19.59 ± 9.71 for HC and 24.32 ± 8.03 for CRC, *p* < .001). In peripheral blood, 25(OH)D level had negative correlation with TGF‐β1 (*r* = −.42, *p* < .001), IL‐10 (*r* = −.36, *p* < .001), IL‐23 (*r* = −.38, *p* < .001), and IL‐17 (*r* = −.27, *p* = .008) levels (Figure 4E–H). IL‐6 is a key player in Th17/Treg balance. ELISA result demonstrated that the level of IL‐6 was significantly higher in CRC patients (*p* < .001) (Supporting Information: Figure S2A). In peripheral blood, 25(OH)D level had negative correlation with IL‐6 level (*r* = −.38, *p* < .001) (Supporting Information: Figure S2B).

### 3.5 The ratio of treg and Th17 cells in CRC patients with different grades of TNM stage

A total of 95 CRC patients in this study was divided into TNM stages of I–II (*n* = 44) and III–IV (*n* = 51). Th17 and Treg cell ratios were significantly upregulated in CRC patients with TNM stages of III–IV (Supporting Information: Figure S3A, *p* = .003; and Supporting Informatin: Figure S3B, *p* = .006). ELISA results demonstrated that the levels of IL‐17, IL‐23, IL‐10, TGF‐β1, and IL‐6 were significantly higher in CRC patients with TNM stages of III–IV (Supporting Information: Figure S3C, *p* = .015; Supporting Information: Figure S3D, *p* = .033; Supporting Information: Figure S3E, *p* = .005; Supporting Information: Figure S3F, *p* = .019; Supporting Information: Figure S3G, *p* = .006).

## 4 DISCUSSION

High 25(OH)D level decreases the incidence and mortality of several malignant cancers, including breast, ovarian, colorectal, rectal, lung, and prostate cancers, and affected their prognosis.^13^ 25(OH)D is the main vitamin D circulating form, with a long half‐life, high and stable concentration in blood, reflecting vitamin D amount consumed and synthesized by oneself as well as the ability of vitamin D conversion. Decreased serum 25(OH)D level triggers CRC development.^14^ An inverse linear relationship is observed between 25(OH)D concentrations and CRC risk.^15^ Higher vitamin D intake can reduce CRC incidence by 20%–30%.^16^ Studies have found that high vitamin D level can reduced CRC development risk, especially in women over the age of 60.^17^

In CRC patients, 25(OH)D levels were indeed declined, which is consistent with the other research. Using 20 ng/ml as the boundary, CRC patients were divided into two groups. In CRC patients with no metastasis, TNM stage I–II, and poor differentiation, 25(OH)D levels were dramatically higher than in those with lymph node metastasis, TNM stage III–IV, and moderate to high differentiation.

Inflammation also participates in the development of CRC.^18^, ^19^ Inflammation can promote tumorigenesis and development by stimulating tumor cell proliferation and the release of inflammatory mediators.^20^ CRC patients have immune dysfunction with a Th1/Th2 immune cell imbalance, drifting mainly toward Th2 cells.^21^ Inflammatory factor levels were elevated in CRC patients and influenced by tumor size, tumor stage, tumor metastasis, and tumor pathological histological staging.^22^ Vitamin D deficiency is associated with significant variations in peripheral immune cells and the integrated targeted interventions to both vitamin D and immune system would improve the prognosis of CRC patients.^8^

Th17 cells express and secrete IL‐17, which is involved in regulating the infiltrative microenvironment and is associated with inflammation, autoimmunity, and tumors and has the function of clearing tumor cells.^23^ Treg cells are cells with immunosuppressive functions that induce tumor‐specific local immune tolerance and promote tumor growth.^24^ Studies suggest that Treg and Th17 cells participate in prostate cancer and Th17 cells show antitumor effects.^25^ Treg cells increase in breast, ovarian, lung, and pancreatic cancer patients, and the degree of Treg cell increase correlates with the stage and disease course of the tumor.^26^ The peripheral blood levels of Thl7 and Treg cells were elevated in CRC patients. T 25(OH)D levels in CRC patients had inverse correlation with the ratio of Treg and Th17 cells.

Treg and Th17 cell differentiation is regulated by multiple cytokines. When TGF‐B acts alone, activated initial T cells express Forkhead box P3 (FOXP3) and differentiate into Treg cells; IL‐6 and TGF‐B act together, the initial T‐cell phenotype differentiates into Th17 cells.^27^ Th17 cell survival is regulated by IL‐23 and IL‐17.^28^

Vitamin D inhibits T lymphocytes differentiation and proliferation, which in turn inhibits the secretion of IL‐17.^29^ The immunosuppressive function of vitamin D may occur by the downregulation of IL‐6, IL‐17, and IL‐22 expression in CD4 + T cells.^30^ Vitamin D has a promotive effect on Treg, possibly by a mechanism of directly promoting Treg cell differentiation.^29^

Th17‐related IL‐6, IL‐17, and IL‐23 levels in the CRC group were significantly elevated. Treg cell‐associated TGF‐β and IL‐10 were also elevated. Th17 and Treg‐related molecules TGF‐β, IL‐6, IL‐10, IL‐17, and IL‐23 levels in serum had inverse correlation with 25(OH)D level. Furthermore, CRC patients with TNM stages of III–IV also had higher levels of Thl7 and Treg cells and relative cytokine levels than those with TNM stages of I–II.

Our study has some limitations. First, relatively small sample size did not allow us to carry out stratified analyses with abundant statistical power. Caution must be applied, and the hypothesis and findings should be tested with a larger sample size. Second, our study explores the relationship between serum levels of 25‐OH vitamin D levels and Th17 and Treg changes, but the relative molecular mechanism was not explored. The other lymphocytes subgroups such as CD8 + and T helpers and lymphocyte to neutrophil ratio were not investigated in this study, it should be explored in the future work.

## 5 CONCLUSION

Imbalanced Treg and Th17 cell differentiation in CRC patients led to suppression of the body's immune function and promoted colon cancer development. In CRC patients, 25(OH)D affected the levels of Treg and Th17 cells and relative cytokines. However, the interaction between cytokines and immune cells is complex, and the role of 25(OH)D levels in regulating Thl7 and Treg cells in tumorigenesis and progression should be further explored to promote the development of antitumor immunotherapy.

## CONFLICT OF INTEREST

The authors declare no conflict of interest.

## Supporting information

Supplementary information.
