# Supplementary material for: Low serum level of 25‐OH vitamin D relates to Th17 and treg changes in colorectal cancer patients
Source: Immun Inflamm Dis. 2022 Oct 26;10(11):e723. doi: 10.1002/iid3.723 (PMC9597490; doi:10.1002/iid3.723)
Supplement: Supplementary file 1 — Supplementary information. [file IID3-10-e723-s001.docx]

**Supplementary materials**


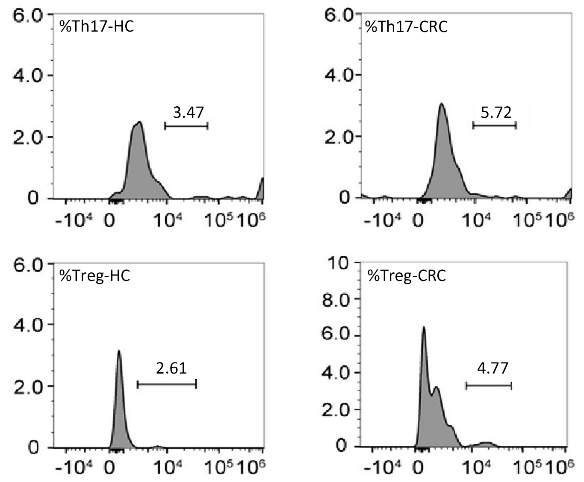


Figure S1. Representative flow cytometry images of Th17 and Treg lymphocyte subpopulations in peripheral blood from healthy controls and CRC patients.


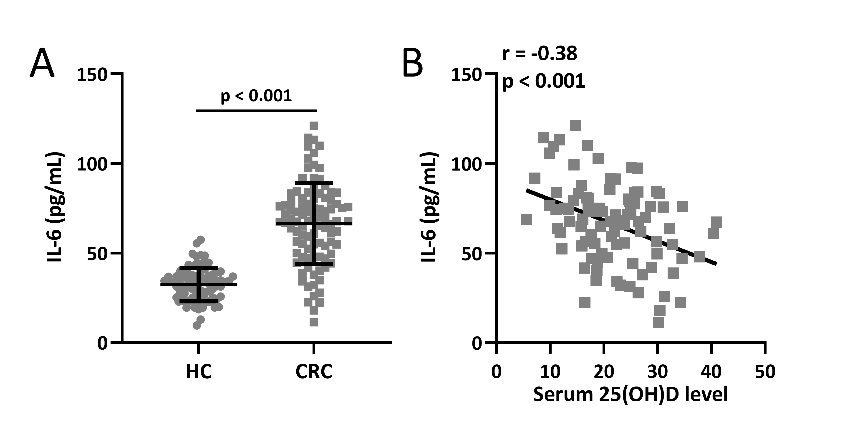


Figure S2. ELISA analysis of IL-6 in serum from healthy controls and CRC patients. n = 80 for HC and n = 95 for CRC. Data were shown as mean ± SD. Mann-Whitney test. The correlation analysis of serum 25(OH)D level and IL-6 in serum from CRC patients, n = 95.


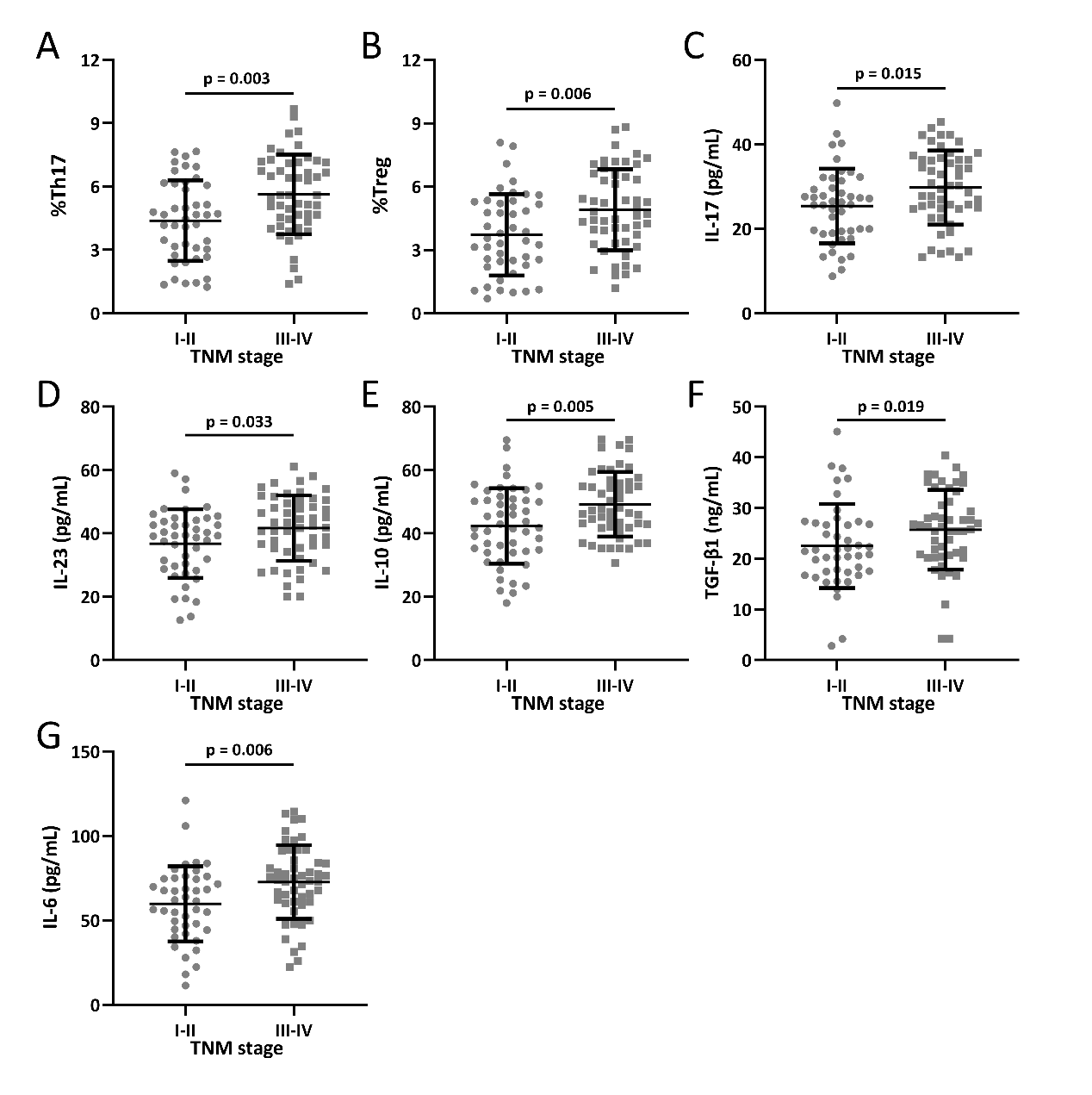


Figure S3. Comparisons of Th17 (A) and Treg (B) lymphocyte subpopulations in peripheral blood, serum IL-17 (C), IL-23 (D), IL-10 (E), TGF-β1 (F) and IL-6 (G) between TNM stages of I-II and III-IV (n = 44 for I-II and n = 51 for III-IV). Data were shown as mean ± SD. Mann-Whitney test.
